# Effects of environmental and physiological covariates on sex differences in unconditioned and conditioned anxiety and fear in a large sample of genetically heterogeneous (N/Nih-HS) rats

**DOI:** 10.1186/1744-9081-7-48

**Published:** 2011-11-25

**Authors:** Regina López-Aumatell, Esther Martínez-Membrives, Elia Vicens-Costa, Toni Cañete, Gloria Blázquez, Carme Mont-Cardona, Martina Johannesson, Jonathan Flint, Adolf Tobeña, Alberto Fernández-Teruel

**Affiliations:** 1 Wellcome Trust Centre for Human Genetics, Roosevelt Drive Oxford, UK; 2 Medical Psychology Unit, Department of Psychiatry and Forensic Medicine, Institut de Neurociències, School of Medicine, Autonomous University of Barcelona, 08193-Bellaterra, Barcelona, Spain

**Keywords:** Anxiety, fear, genetically heterogeneous rats, large samples, environmental covariates, physiological covariates

## Abstract

Physiological and environmental variables, or covariates, can account for an important portion of the variability observed in behavioural/physiological results from different laboratories even when using the same type of animals and phenotyping procedures. We present the results of a behavioural study with a sample of 1456 genetically heterogeneous N/Nih-HS rats, including males and females, which are part of a larger genome-wide fine-mapping QTL (*Quantitative Trait Loci*) study. N/Nih-HS rats have been derived from 8 inbred strains and provide very small distance between genetic recombinations, which makes them a unique tool for fine-mapping QTL studies. The behavioural test battery comprised the elevated zero-maze test for anxiety, novel-cage (open-field like) activity, two-way active avoidance acquisition (related to conditioned anxiety) and context-conditioned freezing (i.e. classically conditioned fear). Using factorial analyses of variance (ANOVAs) we aimed to analyse sex differences in anxiety and fear in this N/Nih-HS rat sample, as well as to assess the effects of (and interactions with) other independent factors, such as batch, season, coat colour and experimenter. Body weight was taken as a quantitative covariate and analysed by covariance analysis (ANCOVA). Obliquely-rotated factor analyses were also performed separately for each sex, in order to evaluate associations among the most relevant variables from each behavioural test and the common dimensions (i.e. factors) underlying the different behavioural responses. ANOVA analyses showed a consistent pattern of sex effects, with females showing less signs of anxiety and fear than males across all tests. There were also significant main effects of batch, season, colour and experimenter on almost all behavioural variables, as well as "sex × batch", "sex × season" and "sex × experimenter" interactions. Body weight showed significant effects in the ANCOVAs of most behavioural measures, but sex effects were still present in spite of (and after controlling for) these "body weight" effects. Factor analyses of relevant variables from each test showed a two-fold factor structure in both sexes, with the first factor mainly representing anxiety and conditioned fear in males, while in females the first factor was dominated by loadings of activity measures. Thus, besides showing consistent sex differences in anxiety-, fear- and activity-related responses in N/Nih-HS rats, the present study shows that females' behaviour is predominantly influenced by activity while males are more influenced by anxiety. Moreover, the results point out that, besides "sex" effects, physiological variables such as colour and body weight, and environmental factors as batch/season or "experimenter", have to be taken into account in both behavioural and quantitative genetic studies because of their demonstrated influences on phenotypic outcomes.

## Introduction

It is well known, from large phenotypic screens, that results of behavioural and physiological/biological phenotyping in rodents are affected by physiological variables, such as sex, weight or coat colour, as well as by environmental variables such as experimenter, testing room/laboratory and season/batch, among others (e.g [1-7]).

Given the multigenic nature of behaviour (e.g [6-8]) as well as of many physiological and disease-related traits, the control of environmental/physiological covariates, as the above mentioned variables, and the assessment of gene-by-environment interactions seems even more important, especially when working with very large samples [6,7,9] as in the case of the present study.

In an excellent QTL (i.e. Quantitative Trait Loci) study of the genetic basis of complex traits in a large sample (n = 2448) of genetically heterogeneous mice, Valdar et al [6,7] demonstrated the existence of many and large gene-by-environment effects (i.e. interactions) on physiological/biological phenotypes, like for example obesity, thermal nociception, immunology, glucose tolerance, and many others [6,7]. Actually, Valdar et al [6,7] study constituted a landmark in the field of quantitative genetics of complex traits, both because its powerful methodological foundations allowing the simultaneous detection and genome-wide fine-mapping of QTLs and because it showed that gene-by-environment interaction effects were even more frequent and larger than the main genetic effects, both on behavioural and on physiological/biological phenotypes. These results have also pointed out the need of mapping the QTLs responsible for these gene-by-environment interactions when aiming to fully understand the underlying mechanisms of the observed phenotypes [6,7,10].

An important concern regarding the aforementioned fine-mapping genetic studies is the type of animals, i.e. the level of genetic recombination. The sample size that is needed to achieve high-resolution fine mapping of QTL, which determines power size as a function of the level of genetic recombination, is another important concern. In this regard, Flint and co-workers [7,11] have demonstrated that simultaneous detection and fine mapping of QTLs is possible by using large samples of genetically heterogeneous animal stocks (see also [12]).

While the above mentioned studies have been carried out in large samples of genetically heterogeneous mice [6,7,10], there is a lack of similar studies in rats. In this context, and within the framework of a European genetic project ("EURATools"; see [13], we are performing the phenotypical characterization of very large samples of genetically heterogeneous N/Nih-HS rats (N/Nih-HS: "*National Institutes of Health Genetically Heterogeneous rat stock"*). We aim to elucidate the genetic and gene-by-environment basis of several unconditioned and conditioned emotionality and anxiety/fear-related phenotypes, as well as the genetic basis of a wide variety of physiological or disease-related phenotypes. All these phenotypes will be submitted to genome-wide fine genetic mapping of QTLs [12,13]. The N/Nih-HS rat stock was formed through an eight-way cross of as much as possible separate inbred strains which were readily available [14]. These eight parental strains were: the MR/N, WN/N and WKY/N (these three strains trace their ancestry to the original Wistar stock); the M520/N and F344/N (both established in the 1920s, but of unknown origin); the ACI/N (hybrid between the August and Copenhagen strains); the BN/SsN (derived from a color mutant from a stock of wild rats kept at the Wistar Institute) and the BUF/N strain [14]. N/Nih-HS rats constitute an ideal tool for fine-mapping of QTLs, as these rats provide very small distance between genetic recombinations [12].

For the present study we have tested N/Nih-HS rats in unconditioned tests for anxiety/fearfulness (i.e. elevated zero-maze and "novel-cage activity" test), as well as for context-conditioned freezing (i.e. conditioned fear to a context) and two-way active avoidance acquisition, which is known to be mediated by a "passive avoidance/active avoidance" conflict which involves conditioned anxiety (e.g [15-18]). Two-way avoidance acquisition was a main phenotype target in the present study, as our previous work has shown that such an anxiety-driven response (e.g [16,17]) appears to have a consistent genetic influence, according to recent QTL studies in rat samples [12,17,19].

Thus we report an initial analysis of sex differences and the effects of environmental independent variables such as batch, season and experimenter, as well as of physiological factors as coat colour or body weight, on unconditioned and conditioned anxiety and fear responses. This analysis should shed light on both future behavioural and genetic (QTL) analyses, with the present type of rats as well as with any other strain.

## Materials and methods

### 2.1. Animals

The subjects were 1456 (698 female and 758 male) N/Nih-HS rats (*"National Institutes of Health Genetically Heterogeneous Rat Stock"*, see [14]; progenitors were kindly provided by Dr. Eva Redei in 2004, Center for Comparative Medicine, Northwestern University, Chicago, USA), females weighing 151 ± 19.7 g (mean ± SD) and males 221 ± 34.2. They were derived from 40 different families which are a breeding colony kept at our laboratory. All litters were culled to 10 pups at birth, trying to keep half of each sex whenever possible. Animals were approximately 8 weeks old at the beginning of behavioural testing. As mentioned above, these rats are part of a high throughput phenotyping protocol in which, besides the behavioural phenotype, a large amount of physiological and disease-related phenotypes are being scored to be submitted to genome-wide fine mapping of QTL (see [12]). Animals were housed in pairs (males) or groups of three (females), in macrolon cages (50 × 25 × 14 cm), and maintained with food and tap water available ad lib, under conditions of controlled temperature (22 ± 2°C) and a 12-h light-dark cycle (lights on at 08:00 h and off at 20:00 h).

### 2.2- Procedure and apparatus

Experiments were performed during the light cycle between 09:00 and 19:00 h, and in accordance with the Spanish legislation on "Protection of Animals Used for Experimental and Other Scientific Purposes" and the European Communities Council Directive (86/609/EEC) on this subject. The experimental protocol was approved by the Autonomous University of Barcelona Ethics committee.

Approximately 2 weeks elapsed between consecutive behavioural tests. Three behavioural tests were administered along a 5-6-week period for each of the 6 batches (with n = 230-270 rats/batch, approximately half of each sex). Phenotyping of the 6 batches was carried out along 2 years (2008-2009). The sequence and the characteristics of the tests were as follows:

#### Elevated zero- maze (ZM)

The maze, similar to that described by Shepherd et al [20] comprised an annular platform (105 cm diameter; 10 cm width) made of black opaque plywood and 65 cm above the ground level. It had two open sections (quadrants) and two enclosed ones (with walls 40 cm height). The subject was placed in an enclosed section facing the wall. The apparatus was situated in a black testing room, dimly illuminated (approximately 50 lux at the level of the apparatus) with red fluorescent light, and the behaviour was videotaped and measured outside the testing room. Latency to enter into an open section (*ZM-LAT*), time spent in the open sections (*ZM-T*), number of entries in the open sections (*ZM-E*), number of stretched attend postures (*ZM-SAP*) and number of defecation boluses (*ZM-D*) were measured for 5 minutes (see [20,21]).

#### Automated novel-cage activity (NACT)

The apparatus (Panlab, Barcelona, Spain) consisted of a horizontal surface (50 × 50 cm) provided with photobeams that detect and measure movement automatically, loading the data in a computer. The subjects were placed in transparent plexiglas cages (40 × 40 × 40 cm). They were situated in a white fluorescent (60 w) illuminated chamber. Spontaneous horizontal activity was measured for 30 minutes, of which we took for analyses the activity scores of the first 5 minutes (*NACT-DIST5; as a measure of novelty-induced -open field-like- activity*) and of the last 5 minutes (*NACT-DIST30*; as a measure of habituated, or less novelty-affected, activity).

#### Two-way active, shuttle box avoidance acquisition (SH) and context-conditioned freezing (fear)

The experiment was carried out with three identical shuttle boxes (Letica, Panlab, Barcelona, Spain), each placed within independent, sound-attenuating boxes constructed of plywood. A dim and diffuse illumination was provided by a fluorescent bulb placed behind the opaque wall of the shuttle boxes, which gave approximately 50 lux intensity inside each of the two compartments of the shuttle boxes. The experimental room was kept dark. The shuttle boxes consisted of two equally sized compartments (25 × 25 × 28 cm), connected by an opening (8 × 10 cm). A 2400-Hz, 63-dB tone plus a light (from a small, 7-W lamp) functioned as the CS (conditioned stimulus). The US (unconditioned stimulus), which commenced at the end of the CS, was a scrambled electric shock of 0.7 mA delivered through the grid floor. Once the rats were placed into the shuttle box, a 4-min familiarization period elapsed before training commenced. Each training trial consisted of a 10-s CS, followed by a 20-s US. The CS or US was terminated when the animal crossed to the other compartment, with crossing during the CS being considered as an avoidance response, and during the US as an escape response. Once a crossing had been made or the shock (US) discontinued, there was a 60-s inter-trial interval (ITI) during which crossings (ITC) were scored. Training consisted of a single 40-trial session.

The variables recorded were the total number of avoidances (*SHAV*), the number of inter-trial crossings (*SHAV-ITC*) and the average response latency for the whole training session (*SHAV-LAT*) (see [15,16]). Context-conditioned freezing was measured by two trained observers (between-observer reliability r = 0.98) as the time a rat spent completely motionless except for breathing movements. Freezing *(FREEZ) *was measured during the first five 60-s inter-trial intervals of the 40-trial acquisition session. No rat made avoidance responses during these first five trials.

### 2.3- Statistical analysis

Pearson correlations and ANOVA analyses, followed by posthoc comparisons when significant (SPSS Windows, 9.0.1, SPSS Inc; USA), were carried out with the most relevant variables from each test. The independent variables/factors included in the ANOVAs were sex, batch (6 levels: each of the 6 groups of 230-270 N/Nih-HS rats of both sexes which were phenotyped during a 3-month period), season (3 levels: spring, fall and winter), experimenter (2 levels -2 experimenters- only applicable to the first test, the non-automated elevated zero-maze test) and colour (5 levels: white, brown, black, brown spotted and black spotted). Body weight (measured before the elevated zero-maze test) was treated as a quantitative covariate and analysed through an ANCOVA test. By using ANOVAs we first aimed at analyzing "sex", "coat colour", "batch" and "season" effects, and the interactions among them regarding influences on the behavioural measures. Secondly, we intended to test "experimenter" effects and its interactions with other independent factors. Finally we wanted to analyze, trough ANCOVA tests, the influence of "body weight" on anxiety and fear dependent variables and whether sex effects were retained regardless "body weight" influence.

Student's t-tests (for independent samples) were also used for between-sex comparisons within batches or within season, provided that we made the *a priori *hypothesis (based on previous and consistent results, as referenced above) that females would be less anxious/fearful than males.

Finally, to study the common factors or dimensions which could underlie the different behavioural tests we performed factor analyses separately for each sex. For the selection of the behavioural variables to be entered in the final factor analyses (i.e. the analyses of the whole test battery) we followed similar criteria to those reported in previous works (see [15,19,22-24]). In short, we applied separate factor analysis with Varimax (orthogonal) rotation to the variables from each test and for each sex separately. These separate factor analyses (Varimax) for each individual test resulted in one factor grouping all the variables (data not shown, in order to save space). Then, in order to select the best variables from the test battery for the final factor analyses, we followed statistical (i.e. choosing variables with the highest loadings) and scientific/empirical criteria, thus also selecting a second variable from each test which was not very related with the first selected one. Following such criteria we know that we are selecting the variables which best represent what the test is measuring and, importantly, we also avoid linear combinations between variables within a given test. Thus, from each of these separate analyses we selected the 2 variables (i.e. 2 variables from each behavioural test/task) which best represented the dimensions or behavioural processes measured by each test. The variables finally selected for the definitive factor analyses which should include the three tests/tasks were: (i) From the elevated zero-maze, ZM-E was selected because it had the highest loading, while the lowest loading was for ZM-SAP, which was also selected because it is an index of anxiety (i.e. it is a "risk assessment" behaviour which is sensitive to anxiolytic and anxiogenic drugs [20,21]) in this test. (ii) From the "novel-cage activity" test, both NACT-DIST5 (lowest loading) and NACT-DIST30 (highest loading) were selected because they represent, respectively, activity in response to novelty and habituated activity. (iii) From the two-way avoidance acquisition session, SHAV-ITC variable showed the highest loading, and FREEZ (lowest loading) was also selected because it represents conditioned fear at the very beginning of the two-way avoidance session (see [24]). These 6 variables, representing the 3 behavioural tests, were then submitted to obliquely-rotated (Oblimin direct) factor analyses to assess the underlying factors that are measured in the behavioural test battery.

## Results

The correlation table (Table 1) shows: 1) high correlations among measures within the same test, especially among those from the elevated zero-maze (r = 0.85 between ZM-T and ZM-E, and 0.40-0.47 among ZM-SAP and the other two variables of the test) and those within the two-way avoidance task (0.51 to 0.68); 2) moderate correlations between context-conditioned freezing (FREEZ) during the first 5 intertrial intervals of the two-way avoidance session and measures of performance in the two-way avoidance task (-0.31 to 0.42); 3) low, although significant correlations (around r = 0.1) between NACT-DIST5 (horizontal exploration/activity in the novel cage during the first 5 minutes) and some of the variables from the elevated zero-maze and the two-way avoidance task, and 4) low but significant correlations among ZM test variables and those from the shuttle box task (e.g. r = 0.11, p < 0.01 between ZM-SAP and SHAV -total avoidance responses-). In order to avoid redundancy we have not included correlations for each sex separately, as their pattern and magnitude were almost identical to those shown in Table 1 for the whole sample of 1456 rats. The present pattern of correlations (sign and magnitude of "r" coefficients) is also similar to that previously observed in a different sample of N/Nih-HS rats (n = 787, approximately half of each sex) which was behaviourally phenotyped in 2005-2006 [19].

**Table 1:** Correlation matrix among the main variables for the whole N/Nih-HS rat sample.

	ZM-E	ZM-T	ZM-SAP	ZM-DEF	ZM-BW	NACT-DIST5	NACT-DIST30	FREEZ	SHAV	SHAV-LAT	ITC	SHAV-ITC
ZM-E	1											
ZM-T	.85**	1										
ZM-SAP	.44**	.41**	1									
ZM-DEF	-.15**	-.16**	-.12**	1								
ZM-BW	-.21**	-.15**	-.04	.22**	1							
NACT-DIST5	.14**	.17**	.18**	-.14**	-.15**	1						
NACT-DIST30	-.01	.03	.02	-.04	-.05	.49**	1					
FREEZ	-.09**	-.05	-.07*	.01	.23**	-.09**	-.01	1				
SHAV	.05	0.5	.10**	.02	-.04	.06*	.03	-.31**	1			
SHAV-LAT	-.14**	-.13**	-.12**	.06*	.37**	-.11**	-.04	.36**	-.47**	1		
ITC	.11**	.12**	.11**	-.04	-.17**	.14**	.05	-.34**	.71**	-.50**	1	
SHAV-ITC	.10**	.11**	.12**	-.03	-.14**	.13**	.05	-.35**	.82**	-.52**	.98**	1

Correlation matrix among the main variables for the whole N/Nih-HS rat sample. Correlations with p ≤.01 and p ≤.001 are shown in bold letters. ZM-E, open section entries (n); ZM-T, time spent (s) in the open sections; ZM-DEF, number of defecation boluses; ZM-SAP, stretched attend postures (n); ZM-BW, body weight right before the ZM test (g); NACT-DIST5, NACT-DIST30 distance (cm) travelled during the first 5 minutes and during the last 5 minutes, respectively, in the "automated novel-cage activity" test; SHAV, avoidances (n) in the shuttlebox; SHAV-LAT, mean response latency (s) in the shuttlebox task; ITC, intertrial crossings (n) in the shuttlebox task; SHAV-ITC avoidances plus intertrial crossings (n) in the shuttlebox task. N = 1456 N/Nih-HS rats of both sexes (see "Materials and Methods"). * p ≤ .01, **p ≤ .001, Pearson's correlation coefficient.

**Figure 1:**
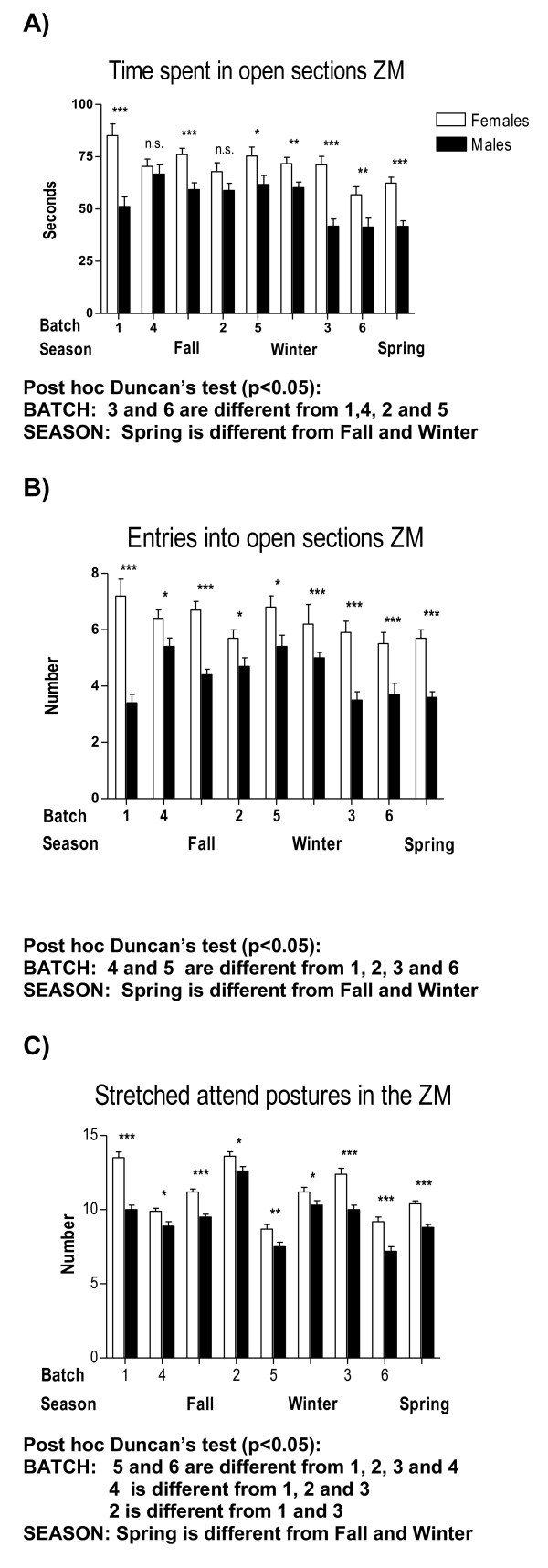
**Between-sex differences in unconditioned anxious behaviour in the ZM test as a function of "batch" and "season"**. For each sex and batch there was a minimum n = 85 rats. Across batches, females were n = 85-144 and males were n = 106-140. *, p < 0.05; **, p < 0.01; **, p < 0.001, between sexes within the same batch (Student's t-tests for independent groups following significant factorial ANOVAs).

**Figure 2:**
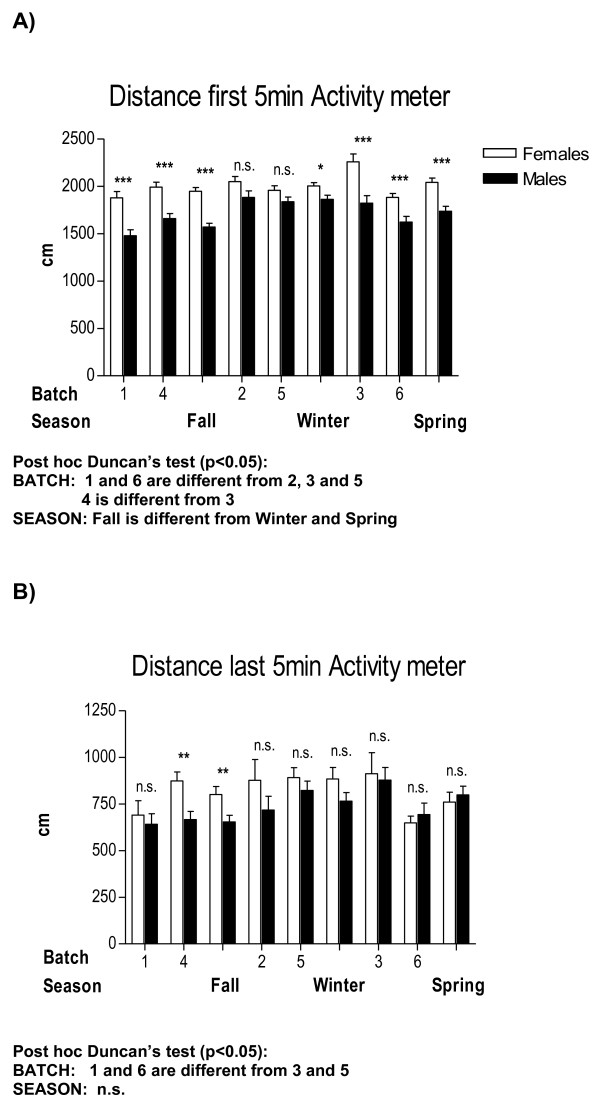
**Between-sex differences in the novel-cage activity test as a function of "batch" and "season"**. All other details as in Figure 1.

**Figure 3:**
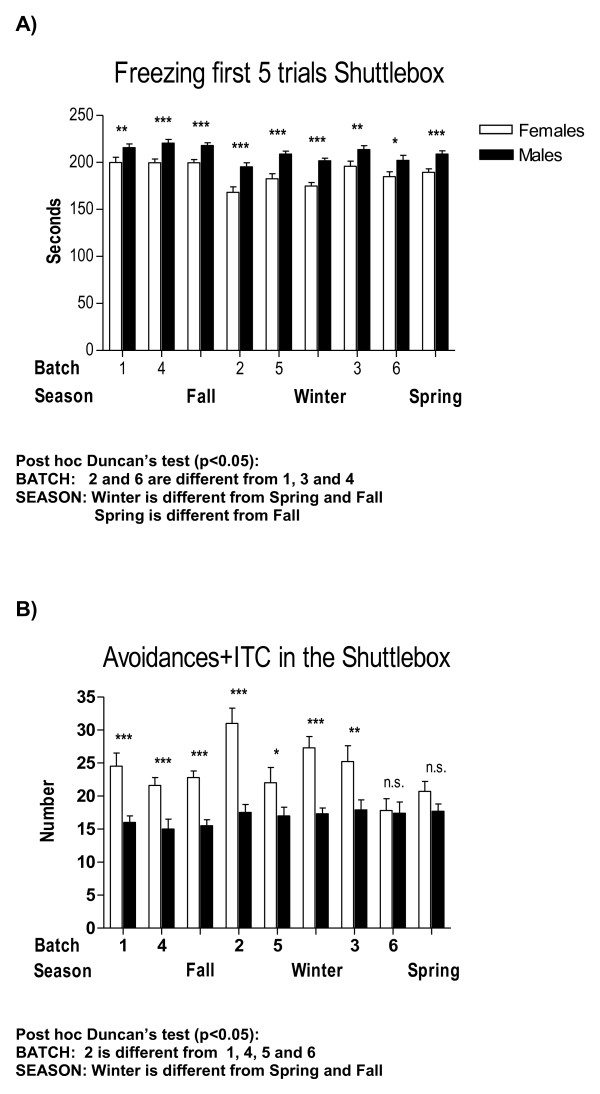
**Between-sex differences in context-conditioned freezing and conditioned anxiety-related responses (avoidances + ITC) in the two-way shuttle box acquisition session as a function of "batch" and "season"**. All other details as in Figure 1.

**Figure 4:**
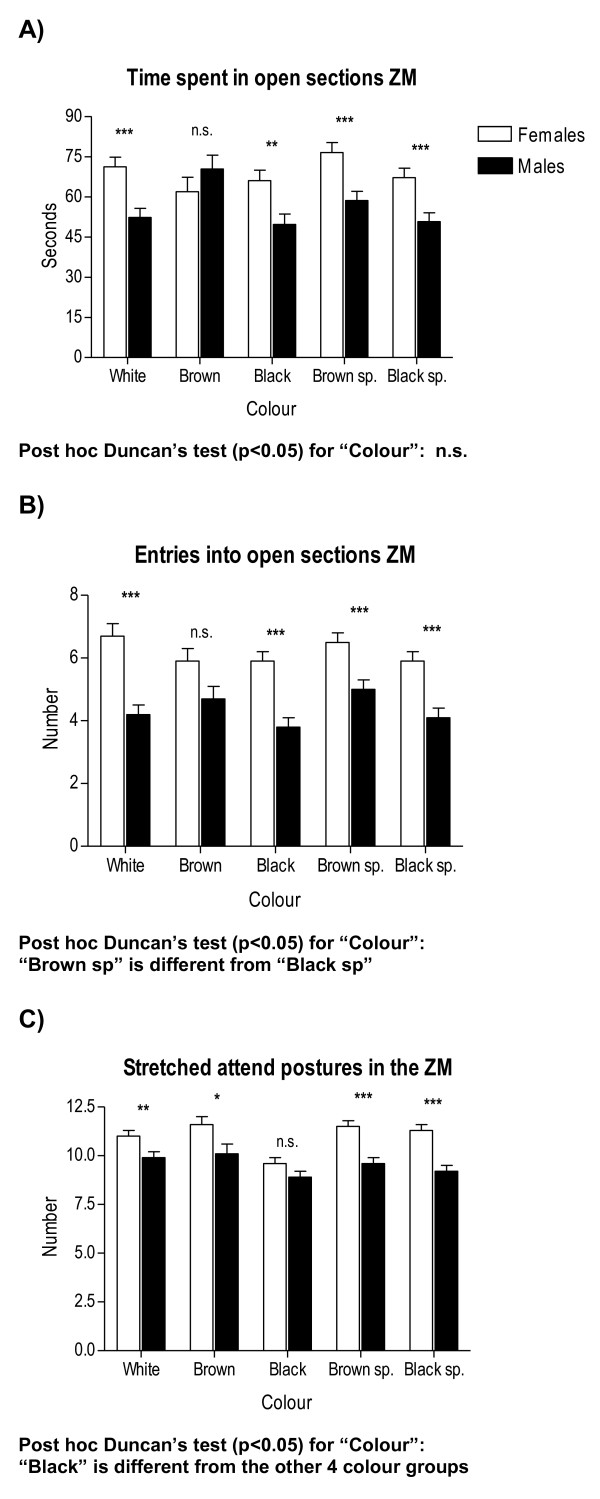
**Between-sex differences in unconditioned anxious behaviour in the ZM test as a function of coat "colour"**. All other details as in Figure 1.

**Figure 5:**
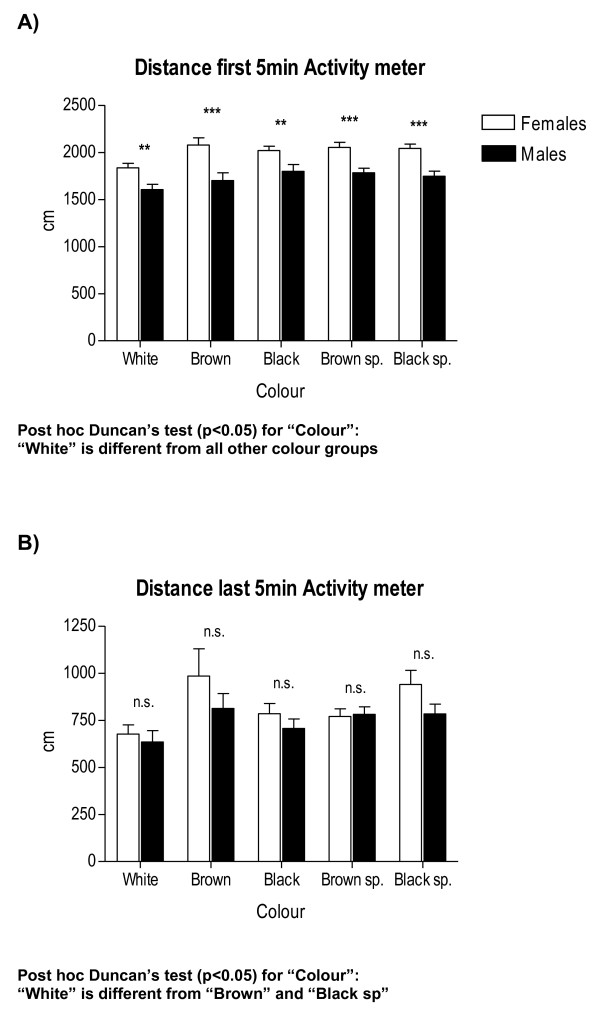
**Between-sex differences in activity in the novel-cage test as a function of coat "colour"**. All other details as in Figure 1.

**Figure 6:**
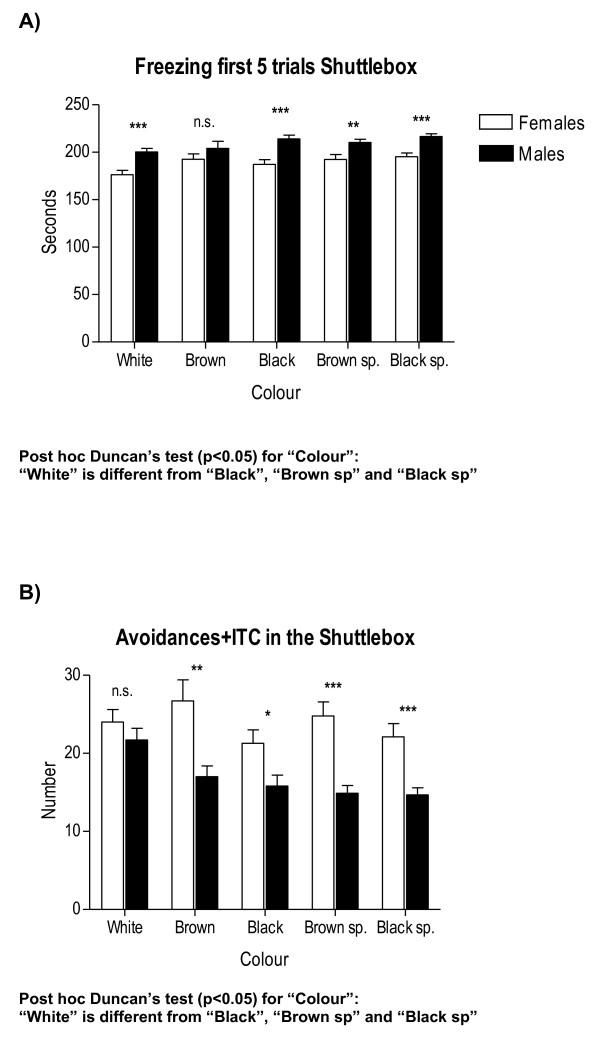
**Between-sex differences in context-conditioned freezing and anxiety-related behaviour (avoidances +ITC) as a function of coat "colour" in the two-way avoidance acquisition session**. All other details as in Figure 1.

**Figure 7:**
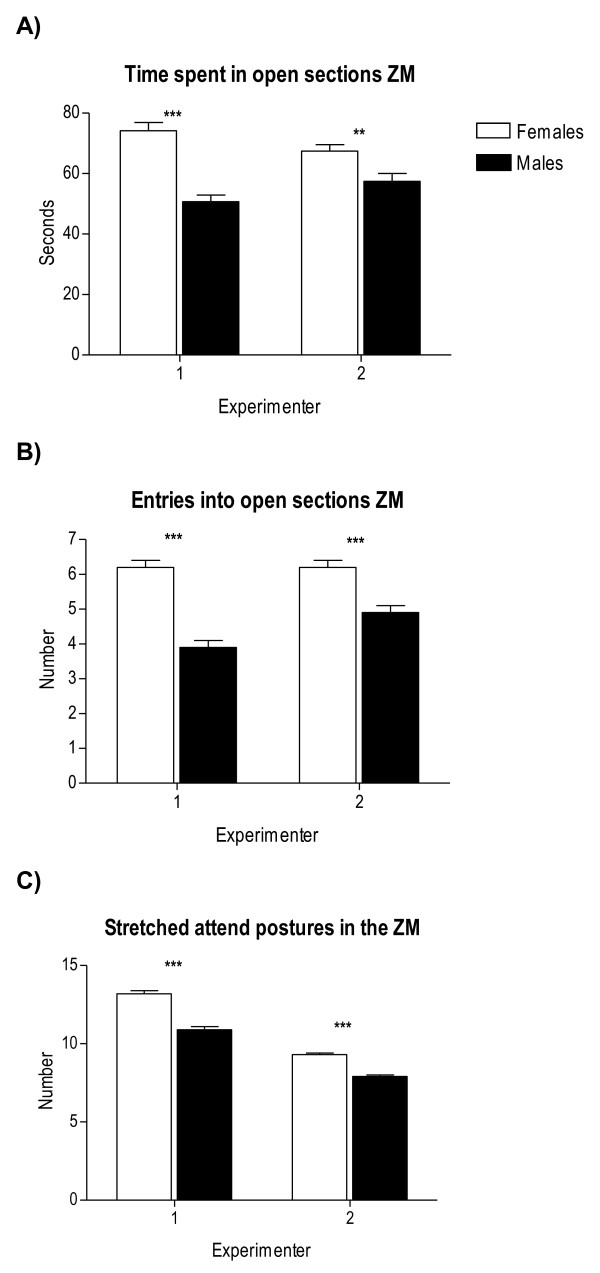
**Between-sex differences in unconditioned anxious behaviour in the ZM test as a function of "experimenter"(see text for "experimenter" statistical effects)**. All other details as in Figure 1.

We have not included a table of descriptives and sex differences for these behaviours, as they can be clearly observed in the figures (Figures 1, 2, 3, 4, 5, 6 and 7).

We first applied four-way ("Sex, 2 levels" × "Colour, 5 levels" × "Batch, 6 levels" × "Experimenter, 2 levels", or "Sex" × "Colour" × "Season, 3 levels" × "Experimenter") ANOVAs to the data. "Batch" and "season" were included in separate ANOVAs because "season" contains "batch" within it. These ANOVAS yielded significant "sex", "colour", "batch", "season" and "experimenter" effects, as well as many significant interactions among these factors. Just as examples of that, if we consider the ZM-T and "avoid+ITC" variables, there were significant effects of "sex" (both variables, F(1, 1427) ≥ 15.3, p < 0.001), "season" (ZM-T variable, F(2, 1427) = 18.4, p < 0.001), "batch" (both variables, F(5, 1427) ≥ 4.5, p < 0.001), "colour" ("avoid + ITC" variable, F(4, 1427) = 5.2, p < 0.001), as well as significant "sex × season" (ZM-T variable, F(2, 1427) = 4.5, p < 0.02), "sex × batch" (both variables, F(5, 1427) ≥ 2.7, p < 0.02), "colour × experimenter" (ZM-T variable, F(4, 1427) = 2.9, p < 0.03), "colour × batch" (ZM-T variable, F(20, 1427) = 1.8, p < 0.02), "sex × colour × batch" (both variables, F(20, 1427) ≥ 1.6, p < 0.05) and "sex × colour × season × experimenter" (both variables, F(8, 1427) ≥ 2.1, p < 0.04) interactions.

Therefore, provided the high number of interaction effects appearing in these initial four-way ANOVA analyses, we have performed specific and separate ANOVAs to test these main factor effects and their interactions in a simpler way. For the sake of clarity, and to save space, Tables 2, 3 and 4 summarize the main and interaction effects of these separate ANOVA analyses for sex differences, also including the physiological (colour) and environmental (batch, season, experimenter) independent factors. Tables 5 and 6 show ANCOVAs including body weight as a quantitative covariate. Instead of using avoidances (SHAV; of which N/Nih-HS rats make on average less than 4 in the whole 40-trial session) as the dependent variable for these analyses, we have used SHAV-ITC (sum of avoidances and intertrial crossings). These two variables are always highly correlated (r = 0.68, Table 1; see also [19]) and it is accepted that ITCs are a kind of "pseudoavoidance" response (they are not related to overall baseline or spontaneous activity, but to actual avoidance acquisition) which highly predicts two-way avoidance acquisition (e.g [15]). Remarkably, main "sex" effects are clear and large on almost all the dependent behavioural variables of the tests (with Fs ranging from 35.0 to 93.3, all p < 0.001; Tables 2, 3 and 4). As shown in Figures 1 (A, B and C), 2 (A), 4 (A, B and C), 5 (A) and 7 (A, B and C), and in agreement with previous studies (using other rat strains or much smaller samples; e.g [19,22]), these sex effects consistently indicate that females show lesser signs of unconditioned anxiety/fearfulness (i.e. see Student's t-tests for ZM-E, ZM-T, ZM-SAP and NACT-DIST5 in Figures 1, 2, 4, 5 and 7) and of conditioned fear/anxiety, as indicated by FREEZ (context conditioned freezing/fear; see Figures 3A and 6A) and SHAV-ITC ("avoidances+intertrial crossings"; see Student's t-tests in Figures 3B and 6B; see also ANOVAs' main "sex" effects in Tables 2, 3, 4, 5 and 6).

**Table 2:** Effects of sex, colour and batch number on ZM, novel cage and shuttlebox variables.

Variable		Sex	Colour	Batch	Sex × Colour	Sex × Batch	Colour × Batch	Sex × Colour × Batch
ZM-E	F = P =	59.6 < .001	2.4 < .05	3.6 < .01	2.4 < .05	4.5 < .001	2.0 < .01	1.9 < .01

ZM-T	F = P =	37.5 < .001	1.9. n.s.	5.5 < .001	1.6 n.s.	3.3 < .01	1.7 < .05	1.9 < .01

ZM-SAP	F = P =	77.0 < .001	3.7 < .01	75.2 < .001	0.9 n.s.	4.0 < .001	2.1 < .01	1.2 n.s.

NACT-DIST5	F = P =	58.0 < .001	3.4 < .01	9.3 < .001	0.1 n.s.	2.6 < .05	1.2 n.s.	1.2 n.s.

NACT-DIST30	F = P =	4.9 < .05	3.5 < .01	3.5 < .01	0.5 n.s.	0.5 n.s.	1.0 n.s.	1.0 n.s.

FREEZ	F = P =	40.3 < .001	4.6 < .001	9.7 < .001	1.0 n.s.	1.6 n.s.	0.7 n.s.	1.7 < .05

SHAV-ITC	F = P =	49.0 < .001	5.2 < .001	4.4 < .001	0.4 n.s.	3.4 < .01	0.8 n.s.	1.6 < .05

Factorial ANOVA analyses, "2 sex × 5 colour × 6 batch", for ZM test, novel-cage activity and shuttlebox variables. For each sex and batch there was a minimum n = 85 rats. Across batches, females were n = 85-144 and males were n = 106-140. See Table 1 for variable symbols.

**Table 3:** Effects of sex, colour and season (of behavioural testing) on ZM, "novel-cage test" and shuttlebox variables.

Variable		Sex	Colour	Season	Sex × Colour	Sex × Season	Colour × Season	Sex × Colour × Season
ZM-E	F = P =	62.0 < .001	2.1 n.s.	6.6 < .001	1.1 n.s.	1.7 n.s.	0.9 n.s.	0.5 n.s.

ZM-T	F = P =	35.0 < .001	2.3 n.s.	13.7 < .001	0.48 n.s.	1.0 n.s.	0.9 n.s.	1.0 n.s.

ZM-SAP	F = P =	42.8 < .001	4.9 < .001	7.6 < .001	1.8 n.s.	1.0 n.s.	1.0 n.s.	1.4 n.s.

NACT-DIST5	F = P =	59.5 < .001	4.4 < .001	10.0 < .001	0.4 n.s.	4.2 < .05	1.2 n.s.	0.6 n.s.

NACT-DIST30	F = P =	5.1 < .05	5.0 < .001	2.7 n.s.	0.8 n.s.	1.7 n.s.	0.7 n.s.	0.6 n.s.

FREEZ	F = P =	51.2 < .001	6.0 < .001	17.2 < .001	0.7 n.s.	1.3 n.s.	0.5 n.s.	2.0 < .05

SHAV-ITC	F = P =	47.7 < .001	3.6 < .01	3.8 < .05	2.5 < .05	4.5 < .05	0.7 n.s.	1.9 n.s.

Factorial ANOVA analyses, "2 sex × 5 colour × 3 season", for ZM test, novel-cage activity and shuttlebox variables. See Table 1 for other details and for variable symbols.

**Table 4:** Effects of sex, colour and experimenter on ZM variables.

Variable		Sex	Colour	Experimenter	Sex × Colour	Sex × Experimenter	Colour × Experimenter	Sex × Colour × Experimenter
ZM-E	F = P =	56.2 < .001	2.6 < .05	4.2 < .05	1.4 n.s.	6.6 < .01	1.1 n.s.	1.2 n.s.

ZM-T	F = P =	36.3 < .001	2.3 n.s.	0.0 n.s.	0.6 n.s.	8.3 < .01	1.0 n.s.	0.8 n.s.

ZM-SAP	F = P =	80.6 < .001	5.4 < .001	314.4 < .001	0.9 n.s.	4.7 < .05	3.5 < .01	0.3 n.s.

Factorial ANOVA analyses, "2 sex × 5 colour × 2 experimenter", for ZM test variables. See Table 1 for other details and for variable symbols.

**Table 5:** Effects of sex and batch number on ZM, "novel-cage test" and shuttlebox variables, taking body weight as a quantitative covariate (ANCOVA analyses).

Variable		Sex	Batch	Body weight	Sex × Batch
ZM-E	F = P =	11.2 < .05	5.4 < .001	6.2 < .001	3.8 < .01

ZM-T	F = P =	6.6 < .001	8.0 < .001	4.9 < .05	3.9 < .01

ZM-SAP	F = P =	30.7 < .001	75.5 < .001	0.3 n.s.	4.5 < .001

NACT-DIST5	F = P =	34.5 < .001	9.5 < .001	1.3 n.s.	2.4 < .05

NACT-DIST30	F = P =	3.0 n.s.	3.9 < .001	0.3 n.s.	1.0 n.s.

FREEZ	F = P =	6.4 < .05	10.5 < .001	5.3 < .05	0.7 n.s.

SHAV-ITC	F = P =	10.3 < .001	4.8 < .001	1.5 n.s.	3.2 < .01

Factorial ANCOVA (analysis of covariance) analysis, "2 sex × 6 batch", including "body weight" as a quantitative covariate, for ZM test, novel-cage activity and shuttlebox variables. See Table 1 for other details and for variable symbols.

**Table 6:** ANCOVA analyses, separating males and females, for the effects of batch on behavioural variables from each sex, after taking "body weight" as a quantitative covariate.

(A) MALES			
**Variable**		**Batch**	**Body weight**

ZM-E	F = P =	7.0 < .001	5.0 < .05

ZM-T	F = P =	7.1 < .001	3.2 n.s.

ZM-SAP	F = P =	37.2 < .001	0.7 n.s.

NACT-DIST5	F = P =	6.3 < .001	0.8 n.s.

NACT-DIST30	F = P =	2.4 < .05	0.4 n.s.

FREEZ	F = P =	4.9 < .001	4.1 < .05

SHAV-ITC	F = P =	0.5 n.s.	2.0 n.s.

(B) FEMALES			

Variable		Batch	Body weight

ZM-E	F = P =	2.7 < .05	1.6 n.s.

ZM-T	F = P =	5.0 < .001	1.8 n.s.

ZM-SAP	F = P =	40.7 < .001	0.3 n.s.

NACT-DIST5	F = P =	5.3 < .001	0.4 n.s.

NACT-DIST30	F = P =	2.6 < .05	0.0 n.s.

FREEZ	F = P =	5.6 < .001	1.6 n.s.

SHAV-ITC	F = P =	5.3 < .001	0.1 n.s.

ANCOVA (analysis of covariance) analyses, separately for each sex and including "body weight" as a quantitative covariate, for ZM test, novel-cage activity and shuttlebox variables. See Table 1 for other details and for variable symbols.

The only exception is NACT-DIST30 (habituated horizontal activity, during the last 5 minutes of the NACT test, Figure 2B), for which sex effects are more marginal (all Fs ≤ 5.1, p < 0.05).

The influence of "batch" (Table 2) and "season" (Table 3; as "season" contains "batch" within it, they were analyzed in separate ANOVAs) is also outstanding, as both independent variables show very significant (p < 0.001) effects on dependent variables from the three behavioural tests (see especially "batch" effects on ZM-T, ZM-SAP, NACT-DIST5, FREEZ and SHAV-ITC in Table 2 which are mostly replicated in Table 3 by "season" effects). Figures 1, 2 and 3 visually show the variation in sex differences across batches or seasons, and also show how some consistently significant sex differences disappear as a function of season (e.g. sex differences in SHAV-ITC are not significant in "Spring" -Figure 3-, or sex differences in NACT-DIST30 are only significant in "Fall" or in "Batch 4" -Figure 2-, among others).

"Colour" main effects appear also for ZM-SAP (p < 0.001; Table 3), for both NACT measures (p < 0.001, Table 3), FREEZ (p < 0.001, Table 3) and SHAV-ITC (Table 2). Finally, there are some very large "experimenter" effects in ZM-SAP (F > 314.4, p < 0.001; Table 4; see also Figure 7), which are due to the fact that one experimenter systematically scored less SAP than the other (although correlations between both experimenters in measures of the elevated zero-maze, including SAP, were r > 0.95). Experimenter effects were not evaluated for variables from the other two behavioural tests either because these were automated or because several experimenters were always and simultaneously involved in placing the animals in the NACT apparatus (4 cages) and in the shuttle boxes (3 boxes).

ANCOVA results of the influence of the quantitative covariate "body weight" on "sex" and "batch" effects are shown in Tables 5 and 6. Significant, although not large, effects of body weight (Fs > 4.9, p ≤ 0.027) appeared on ZM-T, ZM-E and FREEZ variables (but not on NACT variables nor on SHAV-ITC). Nevertheless, despite these "body weight" influences, "sex" and "batch" independent factors retained their highly significant effects in all the variables except NACT-ACT30 (Tables 5 and 6).

Finally, Table 7 (A and B) shows the results of obliquely-rotated factor analyses for the 6 most relevant behavioural variables representing the three behavioural tests (see criteria for selection of these 6 variables in the "Statistical Analysis" section above). Factor analysis led to two-factor solutions in both sexes, explaining approximately 50% variance (Table 7). It is outstanding that, in males, the first factor grouped unconditioned anxiety (elevated zero-maze; 0.71-0.75 loadings), conditioned fear (FREEZ; loading -0.50) and acquisition of two-way active avoidance (SHAV-ITC; loading 0.44), while the second factor represented locomotor activity (0.86-0.87 loadings; Table 7). Conversely, in females the first factor mainly represented activity measures (0.78-0.82 loadings), with much lower loadings of FREEZ (-0.30) and SHAV-ITC (0.40) variables, and the second factor grouped unconditioned anxiety measures from the elevated zero-maze test (both loadings of 0.85; Table 7).

**Table 7:** Two-factor solutions from obliquely-rotated factor analyses applied to the main variables from the three behavioral tests.

(A) MALES		
	**Factor 1**	**Factor 2**

Elevated "zero-maze"		
Entries into open sections	0.71	-
Number of SAPs	0.75	-
Two-way shuttlebox avoidance conditioning		
Time spent freezing trials 1-5	-0.50	-
Avoid40+ITC40	0.44	-
Automated novel-cage activity		
Distance travelled min 0-5 Distance travelled min 25-30	- -	0.86 0.87
Eigenvalues % of accumulated explained variance:	1.68 28.1	1.41 51.6
Correlation between factors = 0.13		

(B) FEMALES		

	Factor 1	Factor 2

Elevated "zero-maze"		
Entries into open sections	-	0.85
Number of SAPs	-	0.85
Two-way shuttlebox avoidance conditioning		
Time spent freezing trials 1-5	-0.30	-
Avoid40+ITC40	0.40	-
Automated novel-cage activity		
Distance travelled min 0-5 Distance travelled min 25-30	0.82 0.78	- -
Eigenvalues % of accumulated explained variance:	1.62 26.9	1.38 49.9
Correlation between factors = 0.11		

Oblique two-factor solution (Direct Oblimin) with the 6 most relevant variables (from the 3 tests) in male (A) and female (B) N/Nih-HS rats. Only loadings with absolute values ≥ 0.30 are shown.

## Discussion

As shown in our previous work [19], and consistent with the literature (see [22]), females show significantly less signs of unconditioned anxiety/fearfulness and higher exploratory drive than males. Likewise, in variables related to learned anxiety or fear, females also show less signs of behavioural inhibition. Thus these sex differences appear, respectively, in variables or responses supposed to reflect unconditioned anxiety or fearfulness, as these measured in the elevated zero-maze test and in the novel-cage test during the initial five minutes (i.e. the automated novel-cage activity test; see [19,23]), and in conditioned responses in the shuttle box task, i.e. conditioned fear (i.e. context-conditioned freezing during the initial stages of the task) and conditioned two-way avoidance acquisition (as indicated by the SHAV-ITC variable). These tests, particularly the elevated zero-maze and the acquisition of two-way active avoidance, are well-validated measures of unconditioned anxiety and conditioned anxiety/fear, respectively (see [16,19,20,23]). The measure of context-conditioned freezing/fear is also relevant, because similar procedures are used in humans to study "pavlovian" aversive conditioning (even if in human studies the usual dependent variable is not freezing, but for example skin conductance, heart rate changes or startle responses), and because classical aversive conditioning shares common neuroanatomical bases in different species [18,24-26]. Exploration of a novel, open field-like environment (i.e. the "novel-cage" activity test), has been traditionally considered as related to fearfulness (i.e. the lower exploration, the higher the level of fearfulness), a contention which is also supported from our previous work showing associations between activity during 5 minutes in the novel cage and typical anxiety responses in the light-dark test and the elevated zero-maze test (see [19,23]).

Previous results from factor-analytical studies have suggested that females' responses in unconditioned anxiety-related tests (e.g. the elevated plus-maze, the hole-board; see [27], but see also [22]) might be predominantly influenced by locomotor activity, whereas males' behaviour would appear to be more dependent on anxiety. The present factorial results appear to lend support to that contention, as activity measures are those with the highest loadings on the first factor in females, whereas anxiety in the elevated zero-maze, conditioned fear/freezing and shuttle box avoidance acquisition are those loading on the first factor in males. Thus it remains possible that our present sex differences are importantly modulated through these divergences in activity-driven behaviour between females and males, an issue that should be evaluated by using tests or tasks not dependent upon locomotor activity. It is worth pointing, in this context, the finding that females from the N/Nih-HS rat stock and from other strains have been found to be more anxious/fearful than males in tests which do not depend on locomotor activity, such as the baseline acoustic startle response and the context-conditioned acoustic startle response [19,22].

On the other hand, our results clearly demonstrate that the effects of some independent variables or factors (usually analyzed as "covariates" in genetic studies) such as batch, season, coat colour, experimenter and body weight, among others, must be taken into account in experiments with laboratory rats [6,7,28]. It is the first time, however, that this type of study is carried out in a very large sample of genetically heterogeneous N/Nih-HS rats. For instance, regarding "season" and "sex × season" effects (see Table 3), the results indicate that rats tested during spring display relatively higher anxiety/fearfulness in the unconditioned ZM test (less time and entries into open sections; see Figure 1), while rats tested during winter display increased active responses -i.e. better acquisition- in the shuttle box (see Avoidances+ITC in Figure 3). The fact that during a given season, which includes approximately 500 tested rats (of both sexes), the results of sex differences can be different from other seasons, reflecting "sex × season" interactions, is an outstanding fact and is likely to be very relevant in genetic studies. Related to that, the observed "batch" effects, as well as the significant "sex × batch" and "sex × batch × colour" interactions (see Table 2), indicate that different batches of approximately 250 rats, including both sexes, are not expected to behave equally as concerns to between-sex differences. Batch effects and "batch" interactions with other factors are, of course, related to season effects, although they are not the same, as can be observed by comparing "sex × batch" or "sex × season" interaction effects in Table 2 and Table 3.

"Experimenter", as an independent variable or factor, can be of especial relevance when a given test is not fully automated and can significantly influence phenotypic results. This was the case of the elevated zero-maze test in the present study. For this test there were only 2 experimenters involved who, after placing the rat in the apparatus, watched the recorded behaviour of the rat on a TV screen outside the experimental room, and measured it for 5 minutes. As seen in Table 4 significant "experimenter" and "sex × experimenter" effects appeared in almost all cases on the 3 main variables of the elevated zero-maze test for anxiety (see also Figure 7), thus indicating that experimenter effects and interactions have to be considered in genetic studies using large rat samples tested along several years (see also [1,6,7]). "Batch × experimenter" or "season × experimenter" effects could not be tested because the two experimenters were not represented in every batch or season.

Coat colour (see Tables 2, 3 and 4) was a physiological independent variable or main factor in the present study. "Coat colour" showed significant main effects in almost all variables (Tables 2 and 3; Figure 4, 5 and 6), as well as some significant interactions with "batch" (Table 2) and "experimenter" (in the ZM-SAP measure; Table 4). Body weight, which was included as a quantitative covariate in the ANCOVA, showed significant influences on some variables of the elevated zero-maze test and context-conditioned freezing in the shuttle box, although these effects were more important in males (see Tables 5 and 6). Even taking into account these influences of body weight through covariance (ANCOVA) analyses, "sex" and "batch" main factors still retained their significant effects on most of the variables from the three behavioural tests/tasks (as can be seen in Tables 6 and 7).

To sum up, between-sex differences (with females being apparently less anxious/fearful and/or more active than males) are consistent across the different behavioural phenotypes measured, in agreement with previous reports [19,22]. Such consistent between-sex differences hold true in spite of the significant influence of several independent variables such as "batch", "colour", "season", "experimenter" or the covariate "body weight", which lead to frequent "sex × batch", "sex × season", "sex × colour" and "sex × experimenter" significant effects. Other than these systematic main "sex" effects, the predominance of significant effects of temporal factors, as batch or season (and their interactions with "sex"), appears to be especially outstanding, as they might partly reflect the influence of other "hidden" environmental factors, as noted by Valdar et al [7].

In addition, besides the ones considered in the present study, other unidentified factors that account for laboratory-related variability should, ideally, be controlled and included as covariates [2]. Even in studies with inbred strains in different laboratories differences are found, so it could be that gene-by-environment interactions are not being noticed. Genetic analyses performed in different environmental settings may erroneously lead to attributing an identical phenotype to different genes [29]. Gene X environment effects might depend on population structure or on the phenotype measure used [30]. Discrepant findings are largely attributable to differences in the number and frequencies of alleles segregating in outbred populations compared with those in crosses between inbred strains. In a complex quasi-outbred stock (known as the heterogeneous stock, HS [6,7]), as it is the case of the present (N/Nih) HS rats, assuming the QTL allele frequencies remain comparable, the same locus is likely to explain about 2, 5% of the variance [31]. In a fully outbred population, the locus will account for much less [32]. This demonstrates that gene-by-environment interactions are frequent even when using inbred rodent strains, but these interactions are still more common (and explain higher percentage of phenotypic variance) when using genetically heterogeneous stocks, which in turn are currently a unique resource for genome-wide fine mapping of genetic and gene-by-enviroment influences on complex traits.

Importantly, "sex" by itself is a very relevant variable or covariate, although for obvious reasons it has been considered as a main factor (independent variable) in the present study. Both main "sex" effects on behavioural phenotypes and interactions of sex with other variables or covariates were shown by Valdar et al [6,7] in their genome-wide genetic study using HS mice. They also reported that significant gene-by-environment interactions explained higher percentage of variance and were more common than main effects [6].

The present analyses point out that "sex" effects on several anxious/fear responses are often modulated by other independent variables, like batch, season or experimenter, for instance. A genome-wide genetic analysis will be performed on a larger N/Nih-HS rat sample for which a wide range of physiological/biological phenotypes (besides the behavioural ones) have been measured. Apart from the present, other covariates (e.g. "hour of the day", "order of testing within a homecage", "cage number/location", "family", "testing cage/box", "study day", etc) will be taken into account when performing the statistical analysis and modeling including all genetic information.

## Competing interests

The authors declare that they have no competing interests.

## Authors' contributions

RL-A, EM-M and AF-T participated in the conception and design of the study, performed the experiments, carried out the statistical analyses and interpretation of the results as well as the writing of the paper. EV-C, TC, GB and CM-C participated in behavioural testing, in data treatment and analyses and in the discussion of the results. MJ, AT and JF participated in the conception and design of the experiments, in the interpretation of the results and in the writing of the paper.

All authors read and approved the final manuscript.
